# Noninvasive Technique for the Screening and Diagnosis of Oral Squamous Cell Carcinoma

**DOI:** 10.7759/cureus.46300

**Published:** 2023-09-30

**Authors:** Shreyash Borkar, Amit Reche, Priyanka Paul, Anvika Deshpande, Mihika Deshpande

**Affiliations:** 1 Public Health Dentistry, Sharad Pawar Dental College and Hospital, Datta Meghe Institute of Higher Education and Research, Wardha, IND

**Keywords:** diagnosis, dermoscopy, oral brush cytology, liquid biopsy, microfuidic detection, optical detection systems, oral squamous cell carcinoma

## Abstract

Oral squamous cell carcinoma (OSCC) is one of the most common types of malignancy. Squamous cell carcinoma is the second-most prevalent type of cutaneous malignancy after basal cell carcinoma. Biopsy followed by histopathological assessment is the primary basis for assessing squamous cell carcinoma, but nowadays optical non-invasive screening modalities are gaining more importance. There has been an emphasis on implementing relatively quick, affordable, and non-invasive screening methodologies because of various limitations associated with conventional screening techniques, including the encroaching characteristic of the biopsy technique, and the increased price value for treatment. Liquid biopsy, optical detection systems, oral brush cytology, and microfluidic detection, are a few examples of these, each of which has advantages and disadvantages of their own. Dermoscopy is one of the fundamental non-invasive screening techniques used for the examination of cutaneous lesions in clinical practice. Optical coherence tomography and high-frequency ultrasound are considered to be beneficial, particularly for assessing the dimensions of tumors before surgery. The primary site of the lesions, tumor diameter, and the state of the operative borders are some factors that can influence prognosis.

## Introduction and background

One of the most prevalent forms of malignant neoplasm is oral cancer, which has a significant global socioeconomic and clinical impact [1]. The World Health Organization (WHO) research states that oral malignancy has a mortality rate of 45% within five years of being diagnosed [2]. On the contrary, if the cancer is diagnosed earlier in its progression, the rate of survival is between 80-90%. The early assessment of various malignancies becomes challenging to establish due to low social awareness and inadequate screening techniques, which contributes to poor prognosis and reduced life expectancy [3]. Oral malignancy is characterized by gradual beginning, complex assessment, aggressive progression, recurrent metastasis, and debilitating therapy. Around 5% of all malignant lesions associated with the body are head and neck cancers and almost 95% of malignancies of the head and neck are squamous cell carcinomas. Across the world, there are regional variations in the prevalence of oral malignancy. Around 40% of oral squamous cell carcinoma (OSCC) occurs on the floor of the mouth or tongue, and 38% of OSCC occurs on the lower lip on the external surface (solar-related cancers) [4]. Squamous cell carcinoma (SCC) often develops on the bald scalp, face, neck, forearms, and dorsal hands over a context of sun-exposed skin. Invasive biopsy and subsequent histopathological analysis are the primary basis for diagnosing SCC. Currently non-invasive and minimally invasive screening modalities are receiving more consideration.

## Review

Search methodology

**Figure 1:**
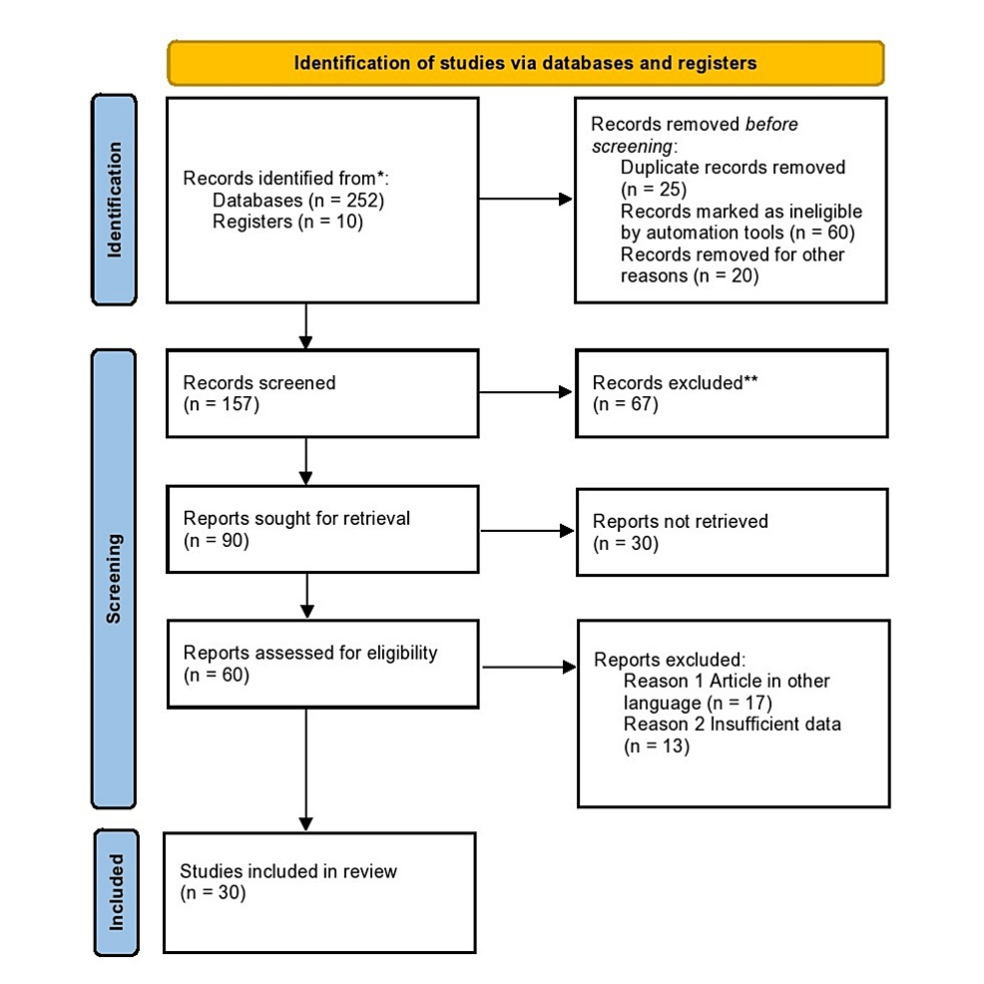
PRISMA flow chart of search strategy PRISMA: Preferred Reporting Items for Systematic Reviews and Meta-Analyses

We conducted a review through PubMed and Google Scholar in July 2022 using keywords such as Oral squamous cell carcinoma (title/abstract), Optical detection systems, Microfluidic detection, Liquid biopsy, and Oral brush cytology. We also searched for key references from bibliographies of the related studies. The search was updated in February 2023. The reviewer monitored the retrieved studies against the inclusion and exclusion criteria in the beginning based on the title and abstract and then on full texts. For inclusion, both published and unpublished studies in the English language were considered. We excluded studies published in other languages because of resource limitations and full-text articles were unavailable to the reviewer. Figure 1 shows the Preferred Reporting Items for Systematic Reviews and Meta-Analyses (PRISMA) flow diagram for the selection process of the article.

Etiology of oral squamous cell carcinoma

There are numerous risk factors that contribute to the occurrence of OSCC such as tobacco, areca nut, alcohol, viruses, and associated family history of OSCC. Alcohol consumption and cigarette smoking are considered to be the main risk factors as those who smoke low- or medium-tar cigarettes and high-tar cigarettes have a higher chance of developing oral malignancy than those who do not smoke [5]. Alcoholic drinks with nitrosamine, urethane content, and ethanol may contain carcinogens or procarcinogens. Alcohol dehydrogenase and cytochrome P450 both help to break down ethanol into acetaldehyde, which has the potential to cause cancer. The diet or nutrition plays an important protective role against the disease as those who eat citrus fruits and vegetables high in beta-carotene have a strong preventive impact against oral cancer.

Various studies demonstrated that socioeconomic factors such as poor socioeconomic status are linked with an increased chance of oral malignancy due to a lack of health education and health services [6]. In certain cases, Candida albicans and viruses including the papillomavirus and herpes virus are considered causative agents of benign and malignant oral lesions. Oropharyngeal malignancies are linked specifically to human papillomavirus [7]. Betel quid is reported as an important etiological factor of oral submucous fibrosis. Chewing betel quid was listed as a significant potential cause of cancer by the International Agency for Research on Cancer in 1986. Chewing areca nuts can alter the cytogenetic integrity of the oral epithelium which can lead to oral cancer.

Techniques for screening and diagnosis

Liquid Biopsy

Liquid biopsy is a minimal-invasive screening or diagnostic technique widely used for cancer assessment and patient stratification [8]. The significance of liquid biopsy in oral squamous cell carcinoma assessment is especially emphasized as this tumor is highly heterogeneous and requires biochemical assessment for effective surveillance and treatment. The liquid biopsy facilitates the assessment of circulating tumor DNA (ctDNA) and released circulating tumor cells (CTCs) in the blood or other body fluids which helps in real-time monitoring of cancer evolution. Various physiological fluids other than blood, which can be implemented for liquid biopsy include urine, salivary secretion, prostatic fluid, hydrothorax secretion, cerebrospinal fluid, mucous secretion, and feces samples [9].

The circulating tumor cells, cell-free DNA, and circulating tumor DNA are a few components that are found in the blood and saliva as biomarkers [10]. The term ctDNA refers to fragmented tumor-derived DNA which is present in systemic circulation and is not linked to any cell, they are an element of circulating cell-free DNA (cfDNA) which implies the total DNA shed into the blood and physiological fluids at the time of apoptosis and necrosis within normal pathological conditions [11]. The ctDNA helps to evaluate the outcomes of therapies and detect relapse earlier. The capillaries receive CTCs discharged from a central tumor, in non-metastatic malignancy these cells are quite rare but can be seen in metastatic carcinomas. In addition to encouraging metastasis, they can infiltrate the main tumor site and assist its development through a mechanism termed tumor self-seeding [12]. Cell Search is the system authorized by the Food and Drug Administration (FDA) for the assessment of breast and bowel malignancies, making it the most widely implemented screening modality for locating and measuring CTCs. Research on people with head and neck SCC showed that people with no disclosed CTCs in their blood had a comparatively better chance of living a healthy life [13]. In addition to effective cancer assessment, management, and recognition of drug resistance liquid biopsy studies can enhance the knowledge of tumor diversity which can help in the advancement of screening modalities.

Light‑Based Detection System

The light-based detection technique is an advanced screening technique that is centered on the optical properties of biological tissues, this diagnostic technique enhances the efficacy of epithelial tissue screening and helps in the diagnosis of various premalignant diseases (PMDs) and OSCC. The concept of optical detection techniques includes chemiluminescence and self-fluorescence imaging. When evaluated by this technique, the variation in the appearance of healthy tissues and pathological lesions is due to the difference in backscattering of light between normal mucosa and tissues with lesions. 

The chemiluminescence is the blue or white illumination (430-580 nm) developed as a result of the chemical interaction between acetylsalicylic acid and hydrogen peroxide within a capsule light rod. The technique is based on the evaluation of tissues with biological changes, such as elevated nuclear/cytoplasmic ratios that reflect light which helps in the detection of lesions. The normal tissues appear darker in contrast to the lesion. The detection system of chemiluminescence marketed under the trade name ViziLite (Zila Pharmaceuticals, Inc., Phoenix, AZ, USA), is a screening system for oral screenings that is designed to empower the better detection and assessment of oral mucosal anomalies in individuals at a greater chance of developing oral malignancy [14].

The mechanism of chemiluminescence refers to the outrush of light with little heat emission (luminescence) [15]. Blue, green, yellow-green, yellow, orange, and red are some colors produced as a result of illumination. There are many different chemiluminescence systems, the two most popular of them are based on the luminol and peroxy-oxalate phenomenon. The peroxy-oxalate system is probably the basis for ViziLite. The theory of chemiluminescence explains that by applying an acetic acid solution the debris is removed and the glycoprotein barrier on the surface epithelium is disrupted as a result, the mucosa is desiccated permitting relatively improved light inflow. As a result, oral mucosal anomalies are better accessed because of variations in the refractive characteristic of the mucosa.

Tissue autofluorescence is a light-based detection technique, it is initiated by the activation of endogenous fluorophores (such as various amino acids, metabolites, and structural proteins) by an external light source [16]. The most significant fluorophores in the oral mucosa are cross-linking collagen in the stroma, nicotinamide adenine dinucleotide (NADH), and flavin adenine dinucleotide (FAD) in the epithelium [17]. The fluorescence occurs when fluorophores absorb photons from outer sources of light and release lower-energy photons [18]. In this technique, the suspected lesion is subjected to monochromatic light as a result fluorescence spectra of endogenous tissue fluorophores are obtained [19]. The cellular changes in the tissue alter the fluorophore concentration which leads to variations in scattering and absorption of light. Dysplastic tissues lose their potential to emit fluorescence because of the disruption of the fluorescent pigment distribution and thus look darker in shade as compared to healthy tissues. The VELscope® (Apteryx Imaging, Atlanta, GA, USA) is a tool that works on the principle of tissue autofluorescence and helps in monitoring the procedure.

Oral Brush Cytology

Oral brush cytology (OBC) is a safe and minimally invasive technique for harvesting cells from the oral mucosa. A toothbrush is used to practice brush cytology, which is simple to perform with minimal cost and discomfort to the patient. This diagnostic technique is helpful when an individual avoids undergoing a biopsy procedure and also when medically compromised patients are subjected to unwarranted surgical risk [20].

This method can be used for early screening of cancerous lesions, in addition to detecting potential biomarkers [21]. Cells from the deeper epithelium layers can be harvested using a cytology brush. A brush specifically developed for oral computer-assisted brush cytology (OralCDx; OralScan Laboratories, Inc., Suffern, NY, USA) is utilized to obtain an entire trans-epithelial specimen. Slides for OralCDx are stained by using a modified Papanicolaou technique. After staining the slides the OralCDx computer system scans them for oral epithelial precancerous and cancerous cells by a neural network-dependent image processing mechanism.

The use of brush cytology which does not require computer assistance is less expensive and can be applicable in locations with limited resources, it is a risk-free way to assess oral lesions [22]. It detects dysplasia in common oral spots that generally do not have any doubtful clinical signs. In brush biopsy cells are collected from the entire thickness of the oral epithelium when compared with exfoliative cytology. Velleuer et al. [23] conducted a statistical examination of 737 lesions, comprising 86 lesions in 30 individuals with at least high-grade oral epithelial dysplasia. The sensitivity and specificity of the OBC technique were 97.7% and 84.5%, respectively, and the DNA ploidy analysis was greater at 100% and 92.2%, respectively. Brush cytology is considered crucial as it helps to intercept misdiagnosing questionable oral lesions or those lesions without a clearly stated etiology, diagnosing major lesions where excision of the complete tissue is not possible or practicable, examining patients with recurrent malignancies, and supervision of premalignant lesions [24].

Optical Coherence Tomography (OCT)

OCT is a light-based detection technique that is based on optical reflection measurements to study cross-sectional views of biological tissues, the technique is implemented for diagnosis and evaluation of oral premalignant lesions (OPMLs) and OSCC [25]. This technique is also known as optical biopsy which offers non-invasive and real-time photographic viewing with a penetration depth of 1.5-3 mm and can also be used to compare images with corresponding histopathological sections. The light interacts with the underlying tissues which helps to evaluate the tissue microstructure up to a certain depth [26]. The OCT imaging is mediated by characteristic variations in tissues that absorb light, whereas in histopathology cellular and subcellular characteristics are studied using particular stains. The OCT is considered a secondary screening technique as it is technique sensitive than the histological screening technique [27]. Low-coherence light is used with a fiber-optic Michelson interferometer in OCT scanning systems to capture 2-dimensional (2-D) and 3-dimensional (3-D) pictures with a resolution of microns-scale from light-scattered biological tissues [28]. The discomfort and inconvenience of a biopsy can be eliminated by using OCT probes to obtain surface and subsurface images of cell structure [29]. This diagnostic technique is considered to be a crucial imaging modality in ophthalmology, the various OCT platforms are FDA-granted for implementation in clinical use [30].

## Conclusions

The article explores various non-invasive techniques for the screening and diagnosis of OSCC. These techniques offer potential advantages over invasive methods like biopsy and histopathological assessment. Screening for timely identification of cancer and pre-cancerous lesions has the ability to lower the morbidity and mortality of this disease. The non-invasive or minimal-invasive technique aims towards an effective outcome of results with minimal expenditure of cost and time. The application of minimally invasive techniques for the detection of cancerous lesions is considered beneficial in various patients where surgical procedures are considered to be a risk factor. The non-invasive screening modalities are considered a valuable tool for real-time monitoring of cancer evolution. The saliva-based detection technique is a new non-invasive method for diagnosing OSCC in the future. Biomedical optics contribute to advanced screening techniques. There are various factors on which the prognosis of disease is dependent which can be assisted by early detection with appropriate screening modalities. The most important component influencing the prognosis of OSCC is lymphatic involvement. The size of the tumor has a significant role in lymphatic involvement and often influences the result of therapy. In terms of prognosis, tumor thickness is more important than tumor size and is intimately linked to lymph node metastases. Screening methods that are non-invasive and simple to use in an outpatient setup must be improved for the early identification of carcinomas.
